# Cardiac rehabilitation based on enhanced external counterpulsation in patients with acute coronary syndrome

**DOI:** 10.3389/fcvm.2026.1677312

**Published:** 2026-02-27

**Authors:** Zhihua Xiao, Zhengyong Zhang, Jia Hu, Weiyan Wang, Tao Yang, Wei Tan, Yaxin Ten, Lirong Cai, Min Feng, Shiquan Ye, Chengwen Yang

**Affiliations:** Department of Cardiology, Santai County People’s Hospital (Affiliated Hospital of North Sichuan Medical College in Santai County), Mianyang, China

**Keywords:** acute coronary syndrome (ACS), enhanced external counterpulsation (EECP), majoradverse cardiovascular events (MACE), MI, cardiac rehabilitation

## Abstract

**Background:**

Enhanced external counterpulsation (EECP) has been proven to be a safe, effective and low-cost non-invasive treatment method to improve cardiac function and hemodynamic characteristics, especially for patients with refractory angina pectoris and chronic heart failure. However, it is currently unclear whether EECP treatment is still effective in improving the long-term prognosis of patients with acute coronary syndrome (ACS).

**Objectives:**

This study aimed to investigate the effect of EECP therapy on major adverse cardiovascular events (MACE) in patients undergoing interventional treatment for ACS.

**Methods and results:**

We conducted a retrospective, controlled trial comparing patients with ACS who received EECP treatment or not from January 2020 to June 2022. A total of 798 patients with ACS who met the study inclusion and exclusion criteria were divided into the No-EECP group (*n* = 583) and the EECP group (*n* = 215) according to whether EECP treatment was performed, and the primary endpoint analysis was performed. The primary endpoint was the first occurrence of MACE (all-cause death, recurrent angina, coronary revascularization, non-fatal myocardial infarction, and stroke) during a median follow-up period of 16.4 months. At 1 year after discharge, 245 patients (30.7%) underwent repeat coronary angiography. Compared with the No-EECP group, the EECP group had a lower percentage of circumflex artery as the culprit vessel (44.1% vs. 23.7%, *p* = 0.001) and multivessel coronary artery disease (38.7% vs. 18.6%, *p* = 0.002). Moreover, patients in the EECP group also had lower Gensini scores (*p* < 0.001). During a median follow-up of 16.4 months, the rate of first MACE was significantly higher in the No-EECP group than in the EECP group [19.9% vs. 7.9%; hazard ratio [HR]: 0.81; 95% confidence interval [CI]: 0.74–0.87; *p* < 0.001]. Among them, compared with patients in the No-EECP group, EECP group had significantly lower incidences of recurrent angina (7.0% vs. 2.8%; HR: 0.83; 95%CI: 0.74–0.93; *p* = 0.024) and coronary revascularization (7.5% vs. 3.3%; HR: 0.84; 95% CI: 0.74–0.94; *p* = 0.028), but no significant differences in the incidences of all-cause death, recurrent myocardial infarction, and stroke.

**Conclusion:**

EECP treatment was associated with a reduction in MACE in ACS patients, driven primarily by lower rates of recurrent angina and coronary revascularization. Meanwhile, EECP treatment showed potential benefits in attenuating coronary artery disease progression. These findings suggest that EECP may be a promising adjunct to postoperative cardiac rehabilitation in ACS patients, though they require validation in prospective randomized trials.

## Introduction

Myocardial infarction (MI) is a serious heart disease, threatening the health of all mankind. The main pathogenesis of MI is that the stenosis or occlusion of coronary artery leads to ischemia and hypoxia of cardiomyocytes, which then leads to acute and chronic loss of cardiomyocytes, and finally leads to heart failure (1). In the past few decades, the improvement of pharmacological, invasive and surgical treatment of cardiovascular disease has led to the increase of life expectancy of patients. Despite the use of multiple drugs and invasive treatments, these patients still have symptoms and functional limitations.

External counterpulsation therapy was first used as a resuscitation tool to support the failed heart more than half a century ago, and it was based on the hemodynamic principle of intra-aortic balloon counterpulsation (2). In the past decades, it has evolved into modern enhanced external counterpulsation (EECP), which has been proved to be a safe, non-invasive, low-cost, well-tolerated and clinically effective physical therapy. A large number of clinical trials have shown that it is suitable for patients with intractable angina pectoris and heart failure (2–4). EECP is safe and effective in the treatment of intractable angina pectoris. The average clinical effective rate is 70%∼80%, which can last for 5 years (5). The effect on patients with chronic angina pectoris can last for several years after the treatment is completed (6–8). It is not only safe for patients with heart failure, but also proved to improve the quality of life and exercise ability, and improve the left ventricular function for a long time (4, 5). Interestingly, EECP therapy has been studied for various potential uses other than heart diseases, such as ischemic stroke, restless leg syndrome, sudden deafness, hepatorenal syndrome, erectile dysfunction and so on (5, 9–11).

A large number of studies have proved that EECP has achieved remarkable therapeutic effect in patients with end-stage coronary artery disease, but the long-term prognosis of patients with ACS after percutaneous coronary intervention is still unclear. The aim of this study was to analyze the effect of EECP on adverse clinical endpoints using clinical data of ACS patients followed up regularly for 16 months.

## Methods

### Study population

We designed this study as a retrospective registry of patients admitted with the diagnosis of acute coronary syndromes (ACS) in Santai County People's Hospital (tertiary medical center with emergency departments) of the North Sichuan Medical College from January 2020 to June 2022 [According to the criteria recommended by European Society of Cardiology Guidelines (12)]. Based on the results of coronary angiography, acute myocardial infarction (AMI) or unstable angina (UA) were enrolled. The patients were followed up for 16 months from the date of determining the ACS. Patients' status was checked from medical records in hospitals and by telephone for patients who had been changes in their condition during the follow-up. The outcome was the MACE included all-cause mortality, recurred angina, nonfatal MI, coronary revascularization, and nonfatal stroke. Investigators undertaking data analysis were masked to treatment assignment for primary end points and 16 months telephone follow-up. The study was reviewed and approved by the Ethics Committee of Santai County People's Hospital. All patients provided written informed consent, and this study was conducted in accordance with the Declaration of Helsinki.

### EECP treatment

The patients in EECP group were lying flat on the treatment bed, and the blood pressure cuff was tightly wrapped around the legs, thighs and hips. The inflation and deflation of the cuff were triggered by the ECG signal of the patients, and were carried out according to the cardiac cycle. The cuff pressure and other parameters were set in accordance with previous literature reports (13, 14). All patients received at least 30 1-hour treatments within 4–7 weeks after PCI. The patients in the No-EECP group were ACS patients who were not treated with EECP in the same period. All the diagnosis and treatment processes of the two groups were carried out in accordance with the standard diagnosis and treatment process of ACS except for EECP treatment. After discharge, all patients were regularly followed up in the outpatient department and adjusted for relevant drugs. Demographic and clinical data are extracted from electronic health records. If a patient had been checked multiple times, only the first measurement result was taken. The coronary angiography report was confirmed blindly by two experienced interventional cardiologists. The analyzed coronary angiography data included severity of coronary artery stenosis (left main artery, left anterior descending artery, circumflex artery, right coronary artery), culprit vessel if applicable.

We use a widely accepted scoring system to assess the degree of coronary stenosis: the Gensini score (15).

### Statistical analysis

The mean ± SD was used to describe the measurement data with a near normal distribution, and the t-test was used for analysis. The median and quartiles were used to describe the measurement data with a non-normal distribution, and the Wilcoxon rank sum test was used for analysis of each group. The proportion and 95% confidence interval (CI) were used to describe the count data, and the chi-square test was used for comparison of each group. We used Kaplan–Meier plots to display the cumulative risk of MACE, and a log-rank test was used to compare groups. The data was analyzed by SPSS 25.0 software (New York, USA). The value of two-sided *P* < 0.05 was considered statistically significant.

## Results

### Patients

**Figure 1:**
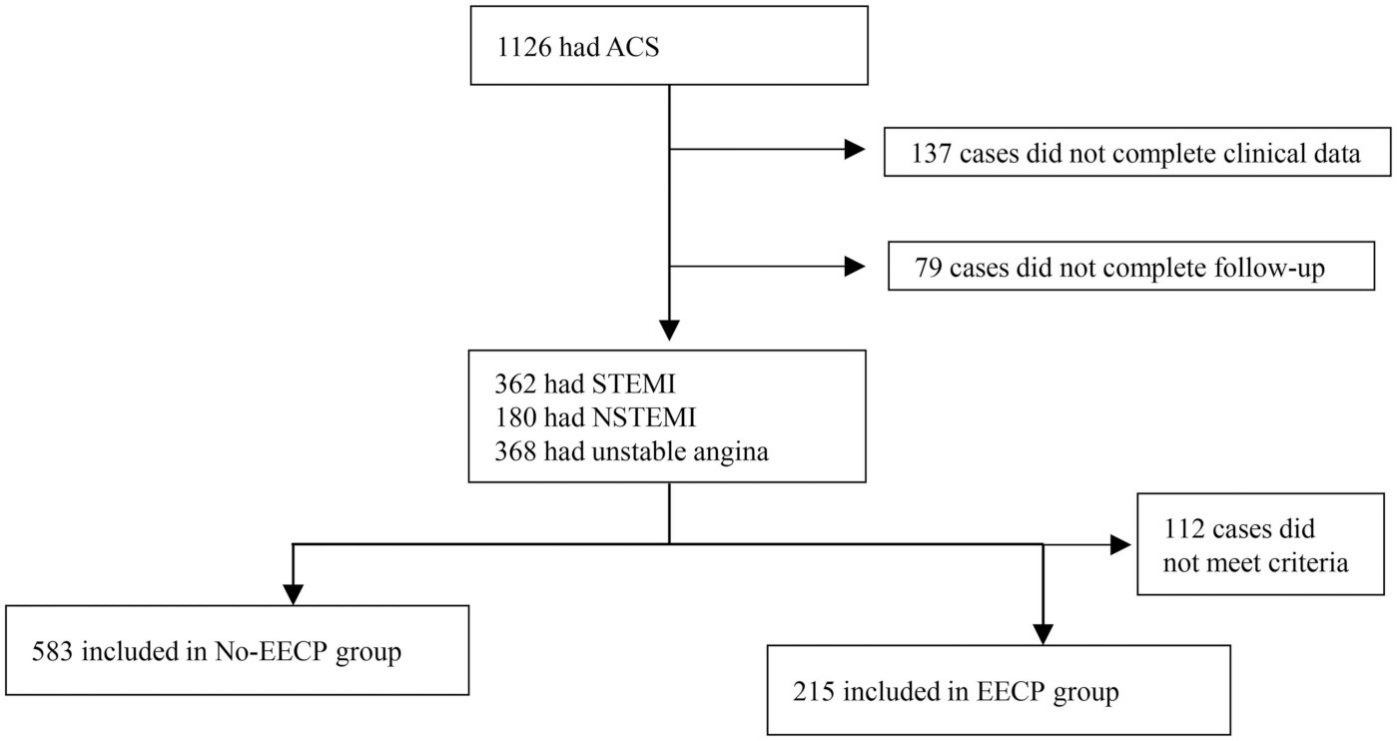
Study profile.

During the period from January 2020 to June 2022, among 1126 patients with ACS who accepted the qualification screening, 137 patients were excluded due to incomplete clinical data and 79 patients’ missing follow-up data. There were 362 patients with STEMI, 180 patients with NSTEMI, and 368 patients with unstable angina pectoris. Finally, 798 patients with ACS (559 males and 239 females; age 68.5 ± 10.2 years) met the inclusion criteria and exclusion criteria. All patients with ACS underwent angiography, 91.4% underwent revascularization, and 26.9% underwent EECP. They were divided into No-EECP group (*n* = 583) and EECP group (*n* = 215) according to whether they were treated with EECP (Figure 1).

The baseline characteristics and vital signs matched well between the two treatment groups (Table 1). There were no differences in age, gender, and body mass index between the two groups of patients. Except for the past medical history of diabetes, hypertension, hyperuricemia, and congestive heart failure, there was no significant difference between the two groups in other past medical history and first medical contact parameters. Compared with the No-EECP group, patients in the EECP group had a higher proportion of history of diabetes (24.9% vs. 35.3%; *p* = 0.003), hypertension (50.4% vs. 60.9%; *p* = 0.008), hyperuricemia (9.3% vs. 20.5%; *p* < 0.001) and congestive heart failure (18.5% vs. 31.6%; *p* < 0.001).

**Table 1:** Baseline characteristics of patients with confirmed ACS.

Characteristic	No-EECP(*n* = 583)	EECP(*n* = 215)
Age, mean (SD), y	69.2 (10.0)	69.7 (10.5)
Male, *n* (%)	405 (69.5%)	154 (71.7%)
BMI, median (IQR), kg/m^2^	24.0 (21.6–26.4)	24.2 (22.8–26.2)
Past history and risk factors, *n* (%)		
Ischemic heart disease	50 (8.6%)	26 (12.1%)
Diabetes mellitus*	145 (24.9%)	76 (35.3%)
Hypertension*	294 (50.4%)	131 (60.9%)
Dyslipidemia*	54 (9.3%)	44 (20.5%)
Congestive heart failure*	108 (18.5%)	68 (31.6%)
COPD	41 (7.0%)	21 (9.8%)
Chronic kidney disease	25 (4.3%)	7 (3.3%)
Stroke	53 (9.1%)	10 (4.7%)
Current or ex-smoker	118 (20.2%)	39 (18.1%)
Current alcohol	56 (9.6%)	18 (8.4%)
Previous PCI	50 (8.6%)	26 (12.1%)
Previous CABG	6 (1.0%)	–
Cancer	15 (2.6%)	4 (1.9%)
First medical contact parameters		
Heart rate, median (IQR), bpm	78 (70–90)	78 (70–90)
SBP, median (IQR), mmHg	134 (118–150)	133 (121–149)
DBP, median (IQR), mmHg	82 (73–91)	83 (73–91)

The data were presented as median and interquartile ranges or percentages, unless otherwise indicated. BMI, body mass index; CABG, coronary artery bypass grafting; COPD, chronic obstructive pulmonary disease; DBP, diastolic blood pressure; IQR, interquartile range; PCI, percutaneous coronary intervention; SBP, systolic blood pressure.

* *P* for difference <0.05.

The procedural details of the two groups of patients are shown in Table 2. The two groups of patients had similar time from arrival to intervention, with a median time of 75.3 min [interquartile range (IQR): 15–190 min] in the No-EECP group and 77.5 min (IQR: 2.4–167 min) in the EECP group (*P* = 0.084). The Gensini scores (No-EECP: 36 [IQR: 20–65.5] vs. EECP: 44 [IQR: 24–71]; *P* = 0.079) of coronary angiography were also similar between the two groups of patients. The surgical details, including the culprit artery, arterial access site, use of thrombus aspiration, and intra-aortic balloon pump, were similar between the two groups. Compared with the No-EECP group, the EECP group had a higher proportion of patients using glycoprotein IIb/IIIa inhibitor (23.0% vs. 29.8%; *p* = 0.009) and drug-eluting stent (90.1% vs. 95.8%; *p* = 0.002).

**Table 2:** Procedural details of patients with confirmed ACS.

Characteristic	No-EECP(*n* = 583)	EECP(*n* = 215)
Hospital arrival to intervention, median (IQR), min	75.3 (15–190)	77.5 (2.4–167)
Length of stay, median (IQR), d	8 (6–10)	8 (7–10)
Cardiac arrest, *n* (%)	26 (4.5%)	11 (5.1%)
Killip class ≥ II, *n* (%)	65 (11.1%)	26 (12.1%)
Gensini score, median (IQR)	36 (20–65.5)	44 (24–71)
Culprit artery, *n* (%)		
LAD	405 (69.5%)	152 (70.7%)
LCX	206 (35.3%)	87 (40.5%)
RCA	282 (48.4%)	113 (52.6%)
Extent of coronary disease, *n* (%)		
Single vessel	308 (52.8%)	101 (47.0%)
Multivessel	258 (44.3%)	109 (50.7%)
LMCA Involvement	23 (3.9%)	6 (2.8%)
Procedural details, *n* (%)		
Radial intervention	556 (95.4%)	206 (95.8%)
Drug-eluting stent*	525 (90.1%)	206 (95.8%)
Glycoprotein IIb/IIIa inhibitor*	134 (23.0%)	64 (29.8%)
Thrombus aspiration	190 (32.6%)	72 (33.5%)
Intra-aortic balloon pump	27 (4.6%)	9 (4.2%)
CABG	12 (2.1%)	–

The data were presented as median and interquartile ranges or percentages, unless otherwise indicated. CABG, coronary artery bypass grafting; LAD, left anterior descending artery; LCX, left circumflex artery; LMCA, left main coronary artery; RCA, right coronary artery; IQR, interquartile range.

* *P* for difference <0.05.

In patients diagnosed with ACS, compared with the No-EECP group, the geometric mean peak value of CK-MB in the EECP group was significantly increased at 27.5 ng/mL [95% confidence interval (CI): 23.9–31.1] vs. 34.1 ng/mL [95% CI: 28.5–39.6], and the ratio of EECP to No-EECP was 1.20 (95% CI: 0.02–2.63; *P* = 0.035). The geometric mean peak values of cTnI, left ventricular internal diameter, and left ventricular ejection fraction were similar between the two groups (Table 3).

**Table 3:** Measures of infarct size in patients with confirmed ACS.

End Point	No-EECP (*n* = 583)	EECP (*n* = 215)	Ratio of means (EECP/No-EECP)	*P* Value
cTnI				
Sample size, n	536	198		
Median peak (IQR), ng/mL	0.12 (0.1–2.1)	0.14 (0.1–4.1)		
Geometric mean peak (95% CI), ng/mL	28.4 (1.8–58.7)	9.6 (1.8–17.5)	0.62 (0.51–0.78)	0.368
Creatine kinase-MB, U/L				
Sample size, n	536	198		
Median peak (IQR), ng/mL	7.4 (2.9–34.0)	10.9 (2.9–67.6)		
Geometric mean peak (95% CI), ng/mL	27.5 (23.9–31.1)	34.1 (28.5–39.6)	1.20 (0.02–2.63)	0.035*
LVID (mm)				
Sample size, n	502	199		
Median peak (IQR), mm	49 (46–54)	49 (46–53)		
Geometric mean peak (95% CI), mm	49.6 (48.7–50.5)	50.4 (49.5–51.3)	1.03 (0.01–2.00)	0.266
LVEF (%)				
Sample size, n	502	199		
Median peak (IQR),	59 (50–63)	58 (50–64)		
Geometric mean peak (95% CI)	55.6 (55.1–56.9)	56.3 (54.9–57.6)	1.02 (0.00–1.00)	0.771
ST-segment elevation	135 (23.2%)	17 (7.9%)	0.78 (0.72–0.84)	<0.001*
T-segment	116 (19.9%)	51 (23.7%)	1.07(0.95–1.19)	0.239

The data were presented as median and interquartile ranges or percentages, unless otherwise indicated. IQR, interquartile range LVID, left ventricular internal diameter; LVEF, left ventricular ejection fraction.

* *P* for difference <0.05.

### Clinical outcomes

407 patients (51%) were reexamined by cardiac ultrasound one year after discharge. The left ventricular internal diameter and ejection fraction were similar in No-EECP group and EECP group. Meanwhile, 245 patients (30.7%) underwent coronary angiography.

The time from discharge to coronary angiography was similar between the two groups. The median time of No-EECP group was 12.3 months (IQR:11.1–13.7 months), and that of EECP group was 12.2 months (IQR:11.2–13.3 months) (*P* = 0.607). Compared with No-EECP group, the percentages of circumflex artery ischemia (44.1% vs. 23.7%, *P* = 0.001) and multivessel coronary artery disease (38.7% vs. 18.6%, *P* = 0.002) in EECP group were lower. In addition, the Gensini score of patients in EECP group (14 vs. 7, *P* < 0.001) was also lower (Table 4).

**Table 4:** Echocardiographic and coronary angiography results of ACS patients 1 year after discharge.

Project	No-EECP (*n* = 583)	EECP (*n* = 215)	*P* Value
Echocardiographic			
Sample size, n	299	108	
First hospitalization to follow-up examination, median (IQR), min	12.1 (9.1–14.2)	11.6 (7.3–12.9)	0.002*
LVID (mm)	49 (46–54)	49 (45–53)	0.867
LVEF(%)	61 (52–65)	61 (52–66)	0.278
Coronary angiography			
Sample size, n	186	59	
First hospitalization to follow-up examination, median (IQR), min	12.3 (11.1–13.7)	12.2 (11.2–13.3)	0. 607
Culprit artery, *n* (%)			
LAD	52 (28.0%)	22 (37.3%)	0.174
LCX	82 (44.1%)	14 (23.7%)	0.001*
RCA	47 (25.3%)	22 (37.3%)	0.074
Extent of coronary disease, *n* (%)			
Single vessel	68 (36.6%)	23 (39.0%)	0.737
Multivessel	72 (38.7%)	11 (18.6%)	0.002*
LMCA Involvement	10 (5.4%)	4 (6.8%)	0.748
Gensini score, median (IQR)	14 (7–25)	7(4–18)	<0.001*

The data were presented as median and interquartile ranges or percentages, unless otherwise indicated. LAD, left anterior descending artery; LCX, left circumflex artery; LMCA, left main coronary artery; RCA, right coronary artery; IQR, interquartile range; LVID, left ventricular internal diameter; LVEF, left ventricular ejection fraction.

* *P* for difference <0.05.

**Figure 2:**
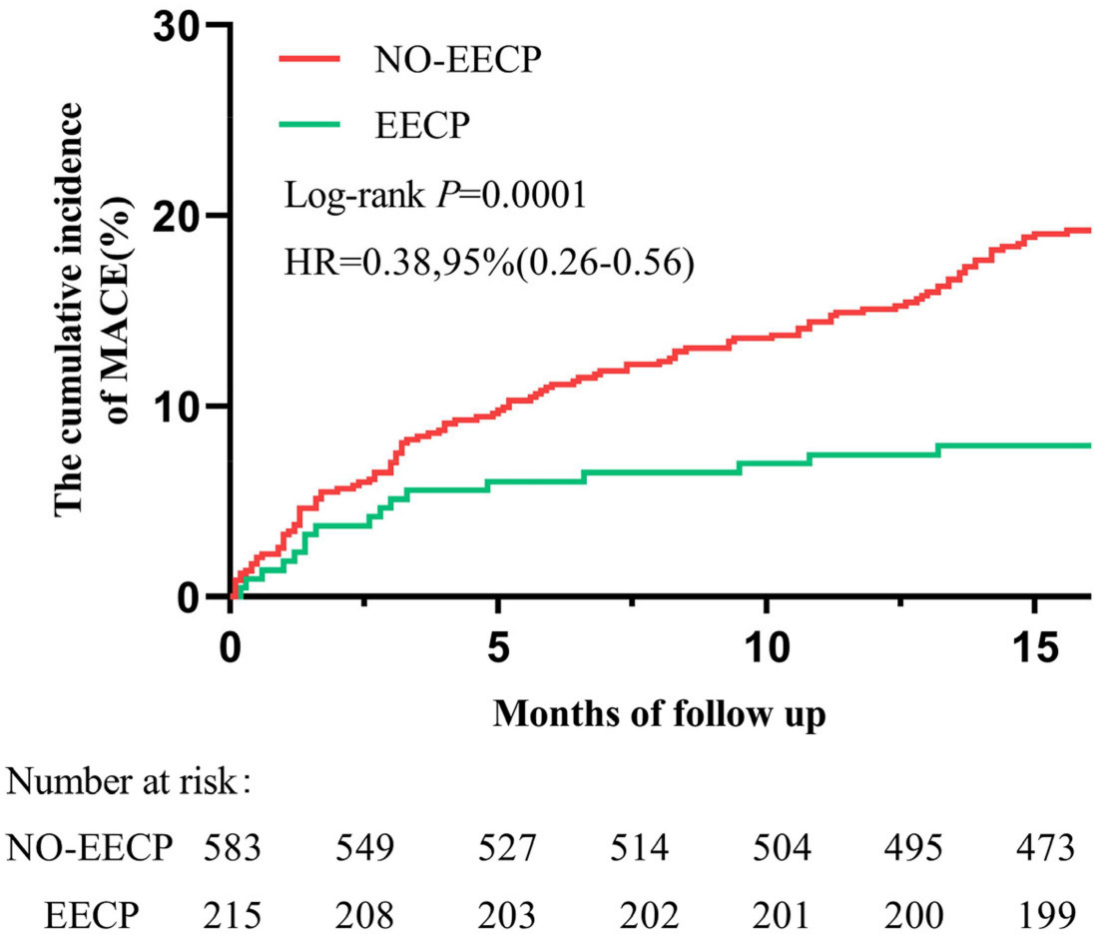
Kaplan–meier plot of cumulative probability of cardiovascular events in No-EECP group and EECP group. MACE, major adverse cardiovascular events.

The safety of the clinical endpoint was monitored 16 months after discharge. During the median follow-up of 16.4 months (IQR:16.1–16.7 months), both groups received appropriate drug treatment. 133 patients (16.7%) had outcomes, including 8 cases (1.0%) of all-cause death, 47 cases (5.9%) of recurrent angina pectoris, 20 cases (2.5%) of non-fatal myocardial infarction, 51 cases (6.4%) of coronary revascularization and 7 cases (0.9%) of stroke. The cumulative first MACE in the No-EECP group were significantly higher than those in the EECP group (19.9% vs. 7.9%; hazard ratio [HR]: 0.81; 95% confidence interval [CI]: 0.74–0.87; *p* < 0.001) (Figure 2 and Table 5). Among them, the incidence of recurrent angina pectoris (7.0% vs. 2.8%; HR:0.83; 95%CI: 0.74–0.93; *P* = 0.024) and coronary revascularization (7.5% vs. 3.3%; HR: 0.84; 95% CI: 0.74–0.94; *P* = 0.028) in the EECP group was significantly lower than that in the No-EECP group. However, there was no significant difference in the incidence of all-cause death, recurrent myocardial infarction and stroke between the two groups (Table 5).

**Table 5:** Adverse clinical end points at the 16-months follow-up in patients with confirmed ACS.

Clinical End Point	No-EECP (*n* = 583)	EECP (*n* = 215)	Hazard ratio (95% CI)	*p* Value
Mortality, any cause	7 (1.2%)	1 (0.5%)	0.83 (0.64–1.09)	0.689
Cardiac cause	5 (0.86%)	1	–	–
Massive hemorrhage	1 (0. 17%)	0	–	–
Cancer	1 (0. 17%)	0	–	–
Recurrent angina	41 (7.0%)	6 (2.8%)	0.83 (0.74–0.93)	0.024*
Non-fatal myocardial infarction	18 (3.1%)	2 (0.9%)	0.81 (0.69–0.94)	0.123
Repeat revascularization	44 (7.5%)	7 (3.3%)	0.84 (0.74–0.94)	0.028*
Stroke or TIA	6 (1.0%)	1 (0.5%)	0.85 (0.63–1.16)	0.681
MACEs	116 (19.9%)	17 (7.9%)	0.81 (0.74–0.87)	<0.001*

The data were presented as percentages, unless otherwise indicated. TIA, transient ischemic attack; MACE, major adverse cardiovascular events.

* *P* for difference <0.05.

## Discussion

We mainly explored the effect of EECP therapy on MACE in patients with ACS after discharge for 16 months. We found that EECP can improve the degree of coronary stenosis and reduce the number of coronary lesions in patients with ACS, but it has no significant effect on left ventricular internal diameter and ejection function. In addition, EECP treatment can significantly reduce MACE, especially recurrent angina pectoris and coronary revascularization.

### Comparison with previous research

Although the life expectancy and quality of life of patients with ACS have been significantly improved in the past few decades, the rehabilitation treatment for patients after discharge is still the largest unsatisfied area (16). External counterpulsation is a safe, noninvasive, low-cost, well tolerated and clinically effective physical therapy, which has been approved by FDA for the treatment of refractory angina pectoris (17). However, the impact of EECP on the long-term prognosis of patients with ACS after interventional therapy is still unclear. Our research results have effectively filled this gap.

Our study found that EECP treatment can improve the Gensini score of coronary angiography, the percentage of culprit vessels in circumflex arteries and multiple coronary lesions in ACS patients, which suggests that EECP treatment can improve the degree of coronary atherosclerosis in patients with ACS. This is consistent with previous reports that low fluid shear stress promotes atherosclerosis, while high physiological shear stress induced by EECP inhibits atherosclerosis and intimal hyperplasia (14). Our results suggest that EECP treatment does not improve left ventricular internal diameter and ejection function. However, a meta-analysis involving 8 randomized controlled trials suggests that EECP can significantly improve the 6-minute walking distance and left ventricular ejection fraction in patients with chronic heart failure (18). The inconsistency with previous research results may be related to the slightly lower proportion of patients undergoing 1-year follow-up cardiac ultrasound (No-EECP group: 51.3%; EECP group: 50.2%).

Our study's follow-up outcomes are similar to those of a multicenter randomized controlled trial of exercise cardiac rehabilitation programs. A total of 85 randomized controlled trials (*n* = 23,430) were included in the recent meta-analysis of exercise-based rehabilitation therapy for coronary atherosclerotic heart disease. During the median follow-up of 12 months, the cardiovascular mortality (RR: 0.74, 95% CI: 0.64–0.86), rehospitalization (RR: 0.77, 95% CI: 0.67–0.89), and recurrent myocardial infarction (RR: 0.82, 95% CI: 0.70–0.96) in the exercise group were significantly reduced, but had no significant effect on all-cause mortality (RR: 0.96, 95% CI: 0.89–1.04) and percutaneous coronary intervention (RR: 0.84, 95% CI: 0.69–1.02) (19). We found that the median follow-up time of ACS patients was 16.4 months, and EECP treatment could significantly reduce the recurrence of angina pectoris (HR:0.83, 95% CI: 0.74–0.93), revascularization (HR:0.84, 95% CI: 0.74–0.94) and MACE HR:0.81, 95% CI: 0.74–0.87). However, EECP treatment has an improvement trend for all-cause death, non-fatal myocardial infarction, and ischemic stroke, but there is no significant difference.

### Potential mechanisms

The mechanism of EECP treatment is mainly focused on patients with refractory angina pectoris or heart failure. It may improve the degree of coronary stenosis and reduce MACE through a variety of mechanisms, including improving endothelial function, promoting collateral circulation, enhancing cardiac function, reducing atherosclerotic burden of arteries and veins, and the effect of peripheral training similar to exercise (5, 20, 21). Early studies have shown that EECP treatment is similar to IABP to maintain or increase the perfusion of heart, lung, brain and other important organs. At present, researchers believe that the therapeutic effect of EECP is closer to exercise training, which significantly improves the central hemodynamics and peripheral endothelial function.

Cardiac ultrasound confirmed the hemodynamic improvement of EECP, providing important theoretical support for the improvement of symptoms in patients with left ventricular systolic dysfunction after receiving EECP treatment. In addition, Gloekler et al. found that EECP can effectively promote the growth of coronary collateral, which is of great significance for improving oxygen supply to ischemic myocardium after myocardial infarction (22). However, in patients with significantly improved exercise tolerance and angina symptoms, radionuclide imaging showed that EECP did not improve myocardial perfusion or ejection fraction, indicating that the mechanisms of increased activity tolerance and clinical improvement are not only central hemodynamic changes, but also other mechanisms.

EECP treatment has a significant impact on the vascular shear stress of the carotid and peripheral arteries (20). Serum VEGF, VEGFR2, and endothelial nitric oxide synthase (eNOS) levels in the treatment were significantly upregulated, while serum Ang2 and endothelin-1 were significantly reduced (13, 23). Correlation analysis shows that the level of vascular active regulatory factors is significantly positively correlated with changes in fluid shear stress in the carotid artery (23). Zhang et al. also found that high physiological shear stress induced by EECP inhibited the proliferation of vascular endothelial cells and smooth muscle cells, thereby improving intimal hyperplasia (22). In addition, Casey et al.'s study showed that EECP treatment improved vascular wall stiffness in both central and peripheral vascular systems (22). These studies have proved that EECP treatment has a direct impact on the peripheral vascular system, which can improve endothelial dysfunction, anti-intimal hyperplasia and atherosclerosis.

In this retrospective analysis, EECP treatment was associated with a reduction in MACE in ACS patients. While the observed associations are promising, it remains difficult to conclusively attribute the improved outcomes to EECP itself. The results of this retrospective analysis should be interpreted as generating the hypothesis that EECP may provide prognostic benefit, which requires direct testing in a randomized controlled trial setting.

### Study limitations

This study has several limitations. First, its retrospective and single-center design may introduce selection bias and unmeasured confounding, such as differences in socioeconomic status, discharge medication adherence, and lifestyle factors, which could influence outcomes. Second, the significant imbalance in group sizes (No-EECP: *n* = 583 vs. EECP: *n* = 215) reflects real-world practice patterns rather than randomization, potentially affecting the statistical robustness and generalizability of the findings. Third, only 51% and 30.7% of the initial cohort underwent follow-up echocardiography and coronary angiography, respectively, introducing potential ascertainment bias. Fourth, we did not assess health-related quality of life or cost-effectiveness, which are important outcomes for rehabilitation therapies. Despite these limitations, baseline and procedural characteristics were well-matched, and the study provides preliminary real-world evidence to inform the design of future prospective trials.

## Conclusions

This retrospective study suggests that EECP treatment may be associated with a reduction in MACE in ACS patients, particularly recurrent angina and coronary revascularization, and shows potential benefits in attenuating coronary artery disease progression. However, due to the observational nature of the study, potential unmeasured confounders, and imbalance in group sizes, a causal relationship cannot be established. These findings should be interpreted as hypothesis-generating. Future multicenter, prospective, randomized controlled trials are warranted to confirm these results and elucidate the underlying mechanisms of EECP in this patient population.

## Data Availability

The original contributions presented in the study are included in the article/Supplementary Material, further inquiries can be directed to the corresponding author.
